# Developing a Suicide Prevention Social Media Campaign With Young People (The #Chatsafe Project): Co-Design Approach

**DOI:** 10.2196/17520

**Published:** 2020-05-11

**Authors:** Pinar Thorn, Nicole TM Hill, Michelle Lamblin, Zoe Teh, Rikki Battersby-Coulter, Simon Rice, Sarah Bendall, Kerry L Gibson, Summer May Finlay, Ryan Blandon, Libby de Souza, Ashlee West, Anita Cooksey, Joe Sciglitano, Simon Goodrich, Jo Robinson

**Affiliations:** 1 Orygen Parkville, VIC Australia; 2 Centre for Youth Mental Health The University of Melbourne Parkville, VIC Australia; 3 The University of Auckland Auckland CBD, Auckland New Zealand; 4 The University of Canberra Bruce, ACT Australia; 5 The University of Wollongong Wollongong, NSW Australia; 6 Portable Collingwood, VIC Australia

**Keywords:** suicide, social media, health promotion, co-design, adolescent, young adult, #chatsafe

## Abstract

**Background:**

Young people commonly use social media platforms to communicate about suicide. Although research indicates that this communication may be helpful, the potential for harm still exists. To facilitate safe communication about suicide on social media, we developed the #chatsafe guidelines, which we sought to implement via a national social media campaign in Australia. Population-wide suicide prevention campaigns have been shown to improve knowledge, awareness, and attitudes toward suicide. However, suicide prevention campaigns will be ineffective if they do not reach and resonate with their target audience. Co-designing suicide prevention campaigns with young people can increase the engagement and usefulness of these youth interventions.

**Objective:**

This study aimed to document key elements of the co-design process; to evaluate young people’s experiences of the co-design process; and to capture young people’s recommendations for the #chatsafe suicide prevention social media campaign.

**Methods:**

In total, 11 co-design workshops were conducted, with a total of 134 young people aged between 17 and 25 years. The workshops employed commonly used co-design strategies; however, modifications were made to create a safe and comfortable environment, given the population and complexity and sensitivity of the subject matter. Young people’s experiences of the workshops were evaluated through a short survey at the end of each workshop. Recommendations for the campaign strategy were captured through a thematic analysis of the postworkshop discussions with facilitators.

**Results:**

The majority of young people reported that the workshops were both safe (116/131, 88.5%) and enjoyable (126/131, 96.2%). They reported feeling better equipped to communicate safely about suicide on the web and feeling better able to identify and support others who may be at risk of suicide. Key recommendations for the campaign strategy were that young people wanted to see bite-sized sections of the guidelines come to life via shareable content such as short videos, animations, photographs, and images. They wanted to feel visible in campaign materials and wanted all materials to be fully inclusive and linked to resources and support services.

**Conclusions:**

This is the first study internationally to co-design a suicide prevention social media campaign in partnership with young people. The study demonstrates that it is feasible to safely engage young people in co-designing a suicide prevention intervention and that this process produces recommendations, which can usefully inform suicide prevention campaigns aimed at youth. The fact that young people felt better able to safely communicate about suicide on the web as a result of participation in the study augurs well for youth engagement with the national campaign, which was rolled out across Australia. If effective, the campaign has the potential to better prepare many young people to communicate safely about suicide on the web.

## Introduction

### Background

Rates of youth suicide are increasing both in Australia [1] and internationally [2,3]. Although young people are often reluctant to seek professional help [4], social media can provide an accepted and accessible platform for them to talk about suicide ideation and can often act as a soft entry point into services [5]. The benefits attributed to social media by young people include the ability to seek help and express feelings in a potentially nonstigmatizing and nonjudgmental environment at any time of the day or night, the capacity to help others, and the sense of community and connection provided [6]. Additional benefits associated with social media include its ability to transcend geographical boundaries, to reach large numbers of often marginalized and hard-to-reach young people, and to deliver preventative interventions quickly and at relatively little cost [6-8].

However, the potential for harm also exists. For example, certain types of content (eg, graphic information or images) can cause distress or may lead to imitative suicidal behavior in others [9]. Furthermore, young people may also be exposed to expressions of suicide risk posted by others but feel ill-equipped to respond. Nonetheless, when it comes to suicide prevention, evidence suggests that social media needs to be viewed as part of the solution, not just part of the problem because of its reach, accessibility, and acceptability [10]. Therefore, interventions that have the capacity to harness the benefits associated with social media, yet can mitigate the potential risks, are required.

Guidelines, which advocate for responsible and sensitive reporting and portrayal of suicide, have improved the safety and quality of communication about suicide in mainstream media [11]. However, although overall suicide reporting guidelines have had good uptake with journalists and appear to be linked to reductions in suicide rates [12], uptake and adherence to guidelines by journalists is not universal [13-15]. Media guidelines also provide little specific advice on using the web-based environment and are unlikely to impact young people as producers of their own content. These limitations highlight that careful consideration needs to be given to implementation strategies and how traditional media guidelines can be adapted for their application to the interactive nature of social media, and so that they reflect the ways young people use these platforms to talk about suicide [16].

In response to this, we have developed the #chatsafe guidelines [17], which were designed to support young people to communicate safely about suicide on social media. To promote awareness, implementation, and dissemination of the guidelines [12], a national suicide prevention social media campaign was developed.

The delivery of population-wide suicide prevention campaigns has gained attention as a potentially effective suicide prevention strategy, and although evidence suggests that they can improve outcomes such as knowledge, awareness, and attitudes toward help-seeking [18-24], evidence supporting their capacity to change behavior is inconsistent, particularly among young people. One explanation for this may be that public health campaigns are rarely co-designed in partnership with the target audience.

The importance of consumer participation, often termed as patient engagement or involvement, user-centered or human-centered design, co-creation, or co-design in health research is becoming increasingly recognized around the world. There has been a significant shift from passive participation, where consumers are merely subjects of the research, toward active participation, where participants have the opportunity to make transformative contributions from the outset. In other industries, user-centered approaches to solving complex problems are widely used [25]. Although studies that actively involve consumers do exist in mental health research, very few involve young people, and fewer still exist in the field of suicide prevention. A recent systematic review identified that out of 99 studies testing suicide prevention interventions among young people, none reported actively engaging young people in the design of the intervention or the research itself [26]. Yet, it stands to reason that an intervention is more likely to achieve its aims if it is co-designed with the people it seeks to assist [25]. However, additional thought needs to be given to the settings, techniques, and materials used in the co-design process when engaging young people and addressing complex and sensitive issues such as suicide [27].

### Objectives

Therefore, to improve dissemination of and engagement with the #chatsafe guidelines, we partnered with young people in the design and development of a social media campaign that aimed to promote safe web-based communication about suicide. The aims of this paper were to (1) describe the co-design process used to develop the #chatsafe campaign strategy; (2) report on young people’s experiences of participating in the co-design workshops; and (3) report on campaign strategy recommendations and describe the final #chatsafe campaign strategy.

## Methods

### Design

A participatory co-design process was employed to generate recommendations for the social media suicide campaign strategy. A total of 11 co-design workshops across 4 Australian states (New South Wales, South Australia, Victoria, and Western Australia) were conducted. Workshops were facilitated by at least two researchers from Orygen and the University of Melbourne (PT, JR, and ML) and two designers or producers from Portable (RB, LS, AW, AC, JS, and SG). The Aboriginal and Torres Strait Islander workshop was led by our Aboriginal researcher (SF) and an Aboriginal consultant. Youth advisors (ZT or RBC) attended 5 of the workshops in a peer capacity. The number of young people participating in each workshop ranged from 6 to 16. Workshops were between 2.5 to 6 hours in duration. The structure and process of these workshops is described in detail as follows.

At the end of each workshop, all young people were invited to complete a specifically designed pen-and-paper quantitative evaluation survey, which was based on one used in an earlier study [10]. The survey included 3 sections: (1) demographics, (2) perceived benefits gained from participation, and (3) workshop acceptability and safety. The survey comprised a combination of dichotomous responses and Likert-type scales and took approximately 10 min to complete.

Given the large number of participants, with multiple small group discussions occurring simultaneously and background music, it was not feasible to audio-record and transcribe workshops. However, a comprehensive postworkshop discussion session, attended by all of the workshop facilitators, occurred within a few days after each workshop to distill key recommendations generated in each workshop. The semistructured discussion involved summarizing the main points that had arisen in the group activities and discussions. These were recorded in detailed notes by RB and were reviewed by all of the relevant facilitators. A total of 11 discussions were held, with 4 to 6 facilitators in each discussion.

### Participants and Setting

The study was conducted by researchers at Orygen and The University of Melbourne, Melbourne, Australia, between December 2017 and May 2019, in partnership with Portable, a digital design and technology company.

Young people aged between 16 and 25 years inclusive, who lived in Australia and were proficient in English, were eligible to participate in the workshops. Lived experience of suicidal ideation or behavior was not an inclusion criterion nor was it an exclusion criterion. Young people were recruited from 7 Australian youth advocacy organizations. Youth program coordinators (or equivalent) distributed advertisements via email lists and Facebook. The advertisement included background information about the #chatsafe project, co-design procedures, reimbursement, and inclusion and exclusion criteria. Interested young people were either referred to PT by program coordinators or contacted PT directly themselves via email to register for a workshop.

One tailored workshop was conducted with Aboriginal and Torres Strait Islander young people at the Second National Aboriginal and Torres Strait Islander Suicide Prevention Conference. Young people who participated in this workshop were recruited via a national bursary award program, which supported travel to and from the conference, accommodation, and conference registration. Young people who were aged between 18 and 25 years inclusive, were an Aboriginal or Torres Strait Islander person, lived in Australia or the Torres Strait, and were proficient in English were eligible to take part.

### Data Analysis

Evaluation data were analyzed using descriptive statistics. Notes from the postworkshop discussions with facilitators were analyzed using a thematic analysis, following a process recommended by Braun and Clarke [28]. We identified a clear analytic question to guide the analysis (ie, how do young people want the #chatsafe guidelines to be implemented via social media?). The written notes from the discussions were then read by PT several times and sorted into initial codes that reflected the diversity of ideas that were generated in the workshops. These codes were then organized into recommendation themes. The notes, codes, and themes were then discussed within the research team to improve the fidelity of the final themes through consensual discussion [29]. The trustworthiness of findings derived from the analysis was established by these discussions. Data trustworthiness was also reached through reflection upon the first author’s biases (PT), through the iterative nature of the co-design workshops and evaluation processes, and by being checked by one of our project youth advisors (ZT) and the designers and producers from Portable (LS, AW, AC, JS, and SG).

### Safety and Ethics

This study received approval from The University of Melbourne Human Ethics Sub-Committee (ID: 1749618) and Western Australian Aboriginal Health Ethics Committee (ID: 887). Written informed consent was obtained in person at the start of each workshop. Parental consent was obtained for young people under 18 years of age. Young people were required to read a *Plain Language Statement* and complete a *Wellness Plan* (see the following paragraph) before the workshop. In line with best practices [30,31], young people were reimbursed for their time.

The *Wellness Plan* represented a collaborative approach to ensuring safety during each workshop. It included personal and emergency contact details, relevant medical details, key contacts (including professional service providers), and self-care and coping strategies for each young person. In instances that a young person became distressed, a researcher accompanied the young person to a private space and enacted their *Wellness Plan*, while the facilitators continued the activity. In addition, a *Risk Management Protocol* was developed, and young people had access to either telephone-based or on-site support from a psychologist during and after each workshop. At the Aboriginal and Torres Strait Islander workshop, there was an on-site Aboriginal psychologist available during and after the workshop.

Finally, the evaluation survey contained 2 safety items: (1) “Did you find participating in this workshop distressing?” and (2) “Would you like to speak about your experiences with a member of the research team?” Young people who responded “yes” to both safety items were asked to provide their contact details. Young people who responded “no” to the second safety item were encouraged to contact youth-friendly telephone and web-based support services. Web and telephone contact details of these services were provided in the survey. Surveys were examined immediately after workshops to identify young people who indicated they wanted support. On-call psychological support was available to the research team via telephone or in-person at all times.

### Structure and Process of Co-Design Workshops

#### Preworkshop Planning Meeting

Approximately 1 week before each workshop, the facilitation team met to clarify roles and review the *Facilitator Resource*. During the meeting, the designers were notified of relevant information from the *Wellness Plans.* Risk mitigation strategies were discussed where appropriate.

#### Workshop Procedure

##### Warm-Up

Each workshop began with a basic introduction activity, during which everyone shared their names, preferred personal pronouns, and one *fun fact* about themselves. This was followed by a warm-up activity and the development of *room agreements*, which involved collaboratively developing some guidelines to ensure everyone felt safe and comfortable during the workshop. In the Aboriginal and Torres Strait Islander workshop, a yarning circle was conducted to open the workshop, and the aforementioned activities were incorporated into this.

##### Co-Design Activities

**Figure 1:**
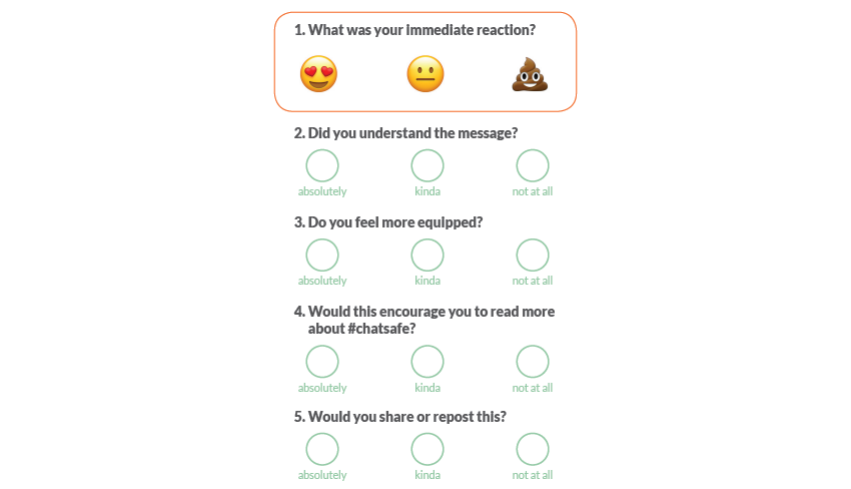
User-testing score card.

The co-design process was iterative whereby the learnings from each workshop informed the schedule of the next one. Although each workshop was unique, they concentrated on the elements of *define*, *design*, and/or *user-testing* (see Textbox 1). Young people were divided into small groups of approximately 4 participants. These smaller groups were facilitated by at least one researcher and one Portable designer. Tools to facilitate design activities were used, such as Scenes (physical and digital illustrations used to develop storyboards; Play Doh; Post-it notes, butcher’s paper, pencils, and colored markers; Lego; stickers; and iPads. At the conclusion of the idea generation segment, the smaller groups presented their ideas to the larger group for discussion. Feedback during user-testing was obtained via group discussions and score cards (Figure 1). Of the 11 workshops, 3 had a *define* focus, 9 had a *design* focus, and 4 had a *user-testing* focus.

During workshops, sensory toys and mindfulness materials were provided, a private break-out area was available, and background music was played to help create a positive environment. Open plan venues were selected to ensure the young people’s safety as they allowed researchers to quickly detect shifts in affect. These types of venues also promoted inclusivity as everyone remained in the same space.

**Co-design element definitions.**

- Define: To map the ways in which young people used social media platforms to communicate about suicide and to determine how young people wanted and needed to see the #chatsafe guidelines implemented via the campaign.
- Design: To develop a social media campaign strategy that incorporated young people’s perspectives and addressed their wants and needs. This included identifying campaign directions (ie, the campaign themes and messages based on the #chatsafe guidelines) and selecting content types (ie, message delivery methods such as video).
- User-Testing: To test and obtain feedback on design prototypes for inclusion in the final campaign.

##### Evaluation and Cooldown

To conclude each workshop, a debrief session was conducted. This provided young people with an opportunity to share their experience, ask questions, and provide feedback. Workshops were closed with a meditation or mood-elevating activity, such as cute animal videos or compliment/appreciation games, where each person provided another person with a workshop-related compliment. In the Aboriginal and Torres Strait Islander group, didgeridoo instrumentals and Australian nature sounds were played. Facilitators remained in the room for at least 10 min after the workshop to provide young people with a private opportunity to ask questions, give feedback, or receive support.

To ensure fidelity across workshops, a *Facilitator Resource* was developed by the project team before the first workshop and updated before each workshop. This included facilitator guidelines, project updates, a workshop agenda, and detailed descriptions of co-design activities for each workshop.

##### Co-Design Barriers and Facilitators

The most significant challenge that we encountered was obtaining ethical approval, which we anticipated given the subject matter of suicide, novel social media component of the project, and the vulnerable populations that we intended to target [32,33]. Unsurprisingly, the Committee was concerned about the safety of participants. To address this concern, detailed and specific written safety protocols (including flowcharts considering the level of risk) were developed to support distressed young people; a clinical psychologist (SR) reviewed the workshop procedures; support from a psychologist was available during workshops; designers and producers were partnered with researchers who were responsible for observing psychological functioning during workshops; and, where possible, designers and producers completed Mental Health First Aid Training to increase mental health literacy and recognize common signs of distress. Given the iterative nature of the project, the Committee were also concerned that they were not reviewing final versions of project procedures and campaign output. To overcome this, a subgroup of the Committee was established to review subsequent project materials as they were produced and made available (eg, workshop agendas and schedules, storyboards, design prototypes, the final campaign strategy, etc). It was agreed that the subgroup would review amendments outside of scheduled meeting dates and give this information immediate attention. In addition to the original application, a total of 11 amendments were submitted and approved.

## Results

The results are divided into the following 2 sections: (1) young people’s demographic characteristics and experiences of the co-design workshops and (2) young people’s content and format recommendations for the #chatsafe social media campaign strategy.

### Participant Characteristics and Evaluation of the Co-Design Process

In total, 134 young people participated in the co-design workshops, of whom 131 (97.7%) completed a pen-and-paper evaluation survey. The characteristics of the young people are summarized in Multimedia Appendix 1.

The majority of young people indicated that they perceived participating in the workshops as a beneficial, acceptable, and safe experience. However, it is important to note that 8 young people reported that the workshop made them feel suicidal and 6 young people were unsure if participation caused them to feel suicidal. Young people’s evaluation of the co-design workshops is reported in Multimedia Appendix 2.

### Recommendations for the #chatsafe Campaign Strategy

A number of key themes were identified that related to both the content and format of the campaign, which informed the final campaign strategy. These have been described in the following sections.

#### Campaign Content

##### Agency and Self-Care

Young people wanted to play an active role in maintaining their own safety. They wanted tools and resources that would equip them to help themselves and their peers. They also wanted the agency and control to choose what content they consumed. Young people highlighted that language used in campaign content should not be too directive. Instead, they wanted prompts that encouraged them to act as *mental health champions* who looked after others as well as themselves.

Young people wanted self-care content to note the importance of checking-in with oneself before supporting others. They liked the idea of talking with a friend, taking a break or going offline, engaging in distraction techniques, and taking control of what they are exposed to on the web (eg, by amending their personal social media settings). Privacy was important to young people, who recommended that the campaign content elicited components of the guidelines that focused on this; for example, content that encouraged private messaging as opposed to public posts when discussing sensitive topics.

##### Stories of Hope and Recovery

Young people reported that real stories of hope and recovery were inspirational, modeled adaptive behavior, normalized and validated challenges, and provided a sense of community. The stories were particularly effective if young people could identify with the protagonist. They also wanted videos that were created by other young people with whom they could relate.

##### Active Support

Young people did not want to self-identify on social media that they needed support. They wanted others, in particular their friends, to be equipped with knowledge and skills to be able to offer support; similarly, they wanted to be able to support their friends. Moreover, communicating with a peer was often reported to be the first step toward receiving professional support. Young people wanted to start with low-impact, informal support options and then build on those, if required.

When discussing the utility of artificial intelligence (AI) on social media, young people wanted AI that was capable of detecting distress, sending personalized information and support, alerting prenominated people, and that was sophisticated enough to engage in natural human dialogue. Some young people from culturally and linguistically diverse (CALD) backgrounds reported that their communities did not talk about suicide, and, in these circumstances, functions such as chatbots could be extremely useful.

Additionally, young people wanted visible links to support services and resources such as web-based and telephone counseling services, as well as the #chatsafe guidelines.

##### Self-Awareness

In the event that a young person’s social media behavior indicated risk, young people reported that they wanted their friends to reach out only if they felt those friends were able to provide support to the young person without causing distress to themselves. They acknowledged that sometimes it could feel overwhelming or beyond their capability to support a friend at risk. As such, they wanted the campaign to include components of the guidelines that promoted *checking-in* with oneself before responding to others.

##### Multicultural Perspectives, Diversity, and Visibility

Young people wanted to feel represented in the campaign. They emphasized that many people identify in multiple ways and had intersectional identities. As a result, they wanted to see a diverse range of young people visibly represented in all content, while simultaneously avoiding tokenism. They wanted nonhuman content to be culturally neutral and gender neutral (eg, anthropomorphic animations).

Although young people wanted the campaign content to be as inclusive as possible, they acknowledged that some ethnic groups required bespoke content, which addressed unique needs in an appropriate, respectful, and meaningful manner. Aboriginal and Torres Strait Islander young people spoke about the shame and stigma associated with suicide in their communities and indicated that they wanted culturally based and community-generated content.

#### Campaign Format

##### Real People

Young people wanted the content to feel realistic and natural. They wanted to see other young people (as opposed to professional actors or models) in videos and photographs. They wanted the content to feel genuine as opposed to staged. They reported that influencers and other brand ambassadors were not relatable in this context unless they were sharing authentic content (eg, personal lived experience).

##### Animation

Animations were considered a popular format for campaign delivery, partly because they were reported to offer cultural protection and prevent overidentification and partly because they were easily distinguishable from other types of content. However, young people still wanted an element of realness, for example, a human voiceover. Conversely, young people stated that mascots, animated animals, memes, and cartoon characters were too flippant in this context and should not be used.

##### Embedded in Existing Social Media Platforms

Young people stated the campaign content had to be available on platforms that they already used, as they did not want to download additional apps, and all tools and resources had to be platform agnostic. They wanted tools and resources to be customizable, adaptive, and interactive. The social media platforms most frequently used were Instagram, Snapchat, YouTube, Messenger, and Facebook. Some young people, particularly those interested in politics or academic research, used Twitter. Lesbian, gay, bisexual, transgender, intersex, queer/questioning, asexual, and other gender or sexual orientation (LGBTIQA+) young people also reported using Discord and Tumblr. They reported that they already used a number of these social platforms to share their experiences and seek support, provide help to others, and remember those who had died, particularly celebrities.

##### Accessibility

As noted earlier, young people wanted the campaign to be as inclusive as possible, and, as such, it was important that the content was accessible to all young people. They stated that video content should include subtitles as well as audio and should be consumable with and without audio. They noted that where voices were used, there should not be more than 2 narrators, so as to prevent confusion. Text-based content should be clear and concise. Many young people, in particular, those with CALD backgrounds, suggested that the content be translated into other languages so that they could share it with their families.

#### Translation of Recommendations Into the Final Campaign

On the basis of the abovementioned findings, a 12-week social media campaign strategy was developed. This comprised 7 campaign directions based on the #chatsafe guidelines: (1) general tips and introduction to #chatsafe; (2) self-care; (3) responding to someone who might be suicidal; (4) what does a safe post look like?; (5) before you post, pause and reflect; (6) remembering someone who has died by suicide; and (7) dealing with harmful content. To avoid overexposure to suicide-related content, every second week, the messaging was focused on self-care. Content relating to suicide of a celebrity was developed but not scheduled for deployment unless a celebrity died by suicide during the #chatsafe campaign (October 2019-January 2020).

Three content types were selected for the campaign: (1) animated videos featuring culturally neutral characters with human voiceovers; (2) photographs or images with text overlay; and (3) videos and photographs featuring real young people as well as pictures, images, and *Boomerangs* produced by young people. A Boomerang video “takes a burse of photos, then speeds them up, and plays them forward and backward to create a looping Boomerang video” [34].

**Figure 2:**
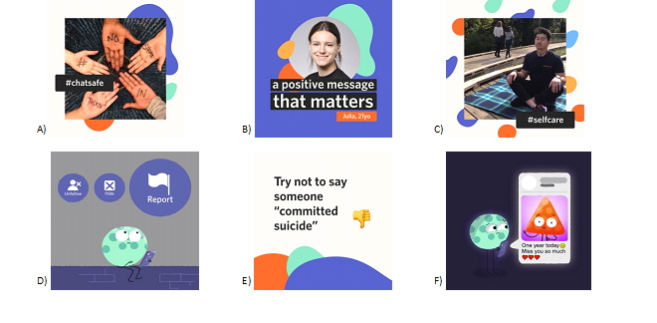
Examples of #chatsafe campaign content. A) General tips and introduction to #chatsafe; B) General tips and introduction to #chatsafe; C) Self-care; D) Dealing with harmful content; E) What does a safe post look like? F) Remembering someone who has died by suicide.

The content was housed on an interactive website (www.orygen.org.au/chatsafe), and the campaign was delivered via Facebook, Instagram, Snapchat, Tumblr, Twitter, and YouTube. Content was deployed 3 times per week over a 12-week period. Each week of the campaign was focused on one of the campaign directions listed earlier. Examples of the campaign content can be seen in Figure 2.

## Discussion

### Principal Findings

This study reported on a participatory approach to co-designing a social media campaign that aimed to help young people communicate safely about suicide on social media. The information contained in the campaign was based on evidence-informed guidelines [17], and campaign information delivery methods were based on the valuable recommendations of the young people involved in the co-design process. The campaign was rolled out across Australia between October 2019 and January 2020. To the best of our knowledge, the #chatsafe campaign was the first of its kind worldwide.

In addition to demonstrating that it was both safe and acceptable to conduct co-design workshops in the field of suicide prevention, young people developed a number of key skills as a result of taking part in the workshops. These included feeling better equipped to communicate safely on the web about suicide and feeling better able to identify others who may be at risk, both of which augur well for the potential impact of the national campaign. Key implications from these findings are discussed as follows.

### Co-Design in Youth Suicide Prevention

Although participatory approaches are becoming increasingly common in mental health [35-43], to the best of our knowledge, they have rarely been applied to the development of youth suicide prevention interventions. One likely explanation for this is fear of iatrogenic effects [44,45], despite the increasing body of literature that suggests that it is both safe and acceptable to conduct suicide research with young people [46-50]. However, there are a few notable exceptions that engage young people in participatory approaches for suicide prevention interventions. Robinson et al [10] engaged secondary school students to develop suicide prevention social media messages. Hetrick et al [51] engaged young people who had experienced depression, suicidal ideation, and self-harm to co-design a mood monitoring app. Neither study reported adverse events, and key benefits identified included improved web-based suicide literacy.

### #chatsafe Co-Design Process

In this study, to mitigate potential risks and to make the activities less intimidating, best practice frameworks for youth participation in mental health and co-design were followed [27,52-58], but a number of modifications to commonly used co-design methods were also made. First, facilitators played a supportive/guiding role rather than a didactic role during design activities [59]. Second, design sprint methods were modified, by adding simple definitions for design terms, providing demonstrations or examples, and removing strict time limits. Finally, detached design personas (ie, stick figures without names, gender, ethnicities, or detailed stories) were used instead of common personas, which may be perceived as too familiar to participants and thereby trigger distress [60].

A multidisciplinary facilitation team comprising mental health researchers and designers and producers conducted the workshops. Designers led the creative activities and the researchers were responsible for the content and well-being of young people. Additionally, 2 project youth advisors attended several workshops in a peer support capacity. Nondiscriminatory language was used at all times [61]. For example, nouns such as *guys* were replaced with gender-neutral alternatives such as *folks*. Finally, open plan venues were used, which not only fostered interactivity but also provided good visibility that allowed the well-being of the young people to be monitored closely.

### Safety

Although we considered and employed robust safety protocols and safety-monitoring techniques, importantly, 8 young people did feel suicidal as a result of the workshops and 6 young people indicated that they were unsure if taking part in the workshop made them feel suicidal. We did not conduct a pre- and postassessment; therefore, we do not know if these young people felt this way before participation. Indeed, the majority of young people who participated in the workshops reported current or previous history of suicidal ideation. Consequently, young people may have been vulnerable to suicidal ideation or may have already experienced suicidal ideation and may have been unsure if the workshop precipitated or perpetuated their thoughts. This illustrates that it may be beneficial for future work to conduct qualitative research on the impacts of participating in the co-design process.

### #chatsafe Campaign Strategy

The co-design activities described earlier led to the development of a suicide prevention social media campaign strategy, in which young people wanted to see specific information on how to help others, positive stories of hope and recovery, and information on youth-friendly sources of support. They wanted to consume this information on the social media platforms that they already used via short and simple shareable and linkable content. Mass media campaigns are becoming increasingly common in suicide prevention [18,62], and social media presents new dissemination opportunities, in particular, for young people who are avid users of social media platforms [63,64]. Despite this, to date, no suicide prevention campaigns exist that specifically target young people, and none have been co-designed with end users.

### Suicide and Social Media

Sharing suicide-related content on social media is often perceived as a double-edged sword [65]. On the one hand, concerns include increased risk of pro-suicide behavior, access to information about suicide methods, fears around contagion, as well as the normalization of suicide behavior [7]. In this study, young people also expressed that exposure to suicidal content can sometimes unwittingly cause distress and that exposure to web-based expressions of suicidal intent can leave them feeling ill-equipped to respond. However, on the other hand, social media can be used to deliver suicide prevention messages to large audiences quickly, to detect intervention opportunities, and to provide users with both formal and informal support [6,9,66]. In this study, young people reported using social media platforms to communicate about suicide in a number of different ways: (1) to share their own experiences of suicidal thoughts and behavior; (2) to support others, in particular their friends; and (3) to discuss and commemorate those who had died by suicide, including friends and family as well as public figures. Thus, given the potential for harm, educating and empowering young people to have these conversations safely about suicide is critical.

### Suicide Literacy

There have been few studies that provide suicide literacy education to young people directly, either on the web or offline. The few offline studies that have been conducted largely focused on relatively small school samples and reported benefits such as improved knowledge, confidence, and capacity to help others [26,67-69]. However, given the proliferation of suicide-related communication on social media platforms, in particular Instagram [70-72], larger scale web-focused studies are required. One recent study by Cheng et al [73] in Hong Kong has attempted this. These investigators cocreated a short suicide prevention video with a YouTube influencer. The video was deployed on both YouTube and Facebook with results showing a promising shift in consumers’ suicide prevention knowledge, attitudes, and willingness to talk about suicide-related feelings [73]. Increasing web-based suicide literacy at the population level in this manner is an important step forward.

### Support Pathways

In this study, young people reported wanting to be actively supported by their friends without having to explicitly state that they needed help. These results are perhaps unsurprising. Young people’s reluctance to actively seek help, and their preference to receive support from their friends as opposed to professionals, is widely documented [4,74-77]. Instead of overtly requesting help, young people will often hint at distress and hope that their peers are equipped to recognize this and respond appropriately [78]; however, this is often not the case. There have been a number of suicide awareness campaigns, but as noted earlier, these appear to have limited capacity to shift behavior [18,19]. This study highlighted young people’s need for interventions that not only increase knowledge and awareness but better equip them to help themselves and each other and they specifically expressed the need for the #chatsafe campaign to provide specific content that would facilitate this. Importantly, young people also identified that talking to their friends on the web about this topic is often a first step toward accessing professional help, hence the need for the campaign to include direct links to professional services.

### Stories of Hope and Recovery

Along with educational content on how to help others, information on where to seek help, and characters whom they could identify with, young people wanted the campaign to contain personal experience narratives that conveyed the stories of other young people who had coped with, or gained mastery over, suicidal ideation. Interestingly, these findings align with the theoretical literature. Resilience-focused content has been shown to have protective effects in studies of mainstream media and has been coined the *Papageno Effect*. The name comes from a character in Mozart’s opera, The Magic Flute, in which Papageno experienced suicidal ideation because he feared he had lost his beloved; however, he refrained from acting on his thoughts because his friends helped him learn alternative coping strategies [79]. Subsequent studies have supported that portrayals of coping with suicidal ideation have protective effects [80-83]. The opposite of the *Papageno Effect* is the *Werther Effect*, which refers to imitative suicides triggered by sensationalist and repetitive communication on suicide. This comes from Goethe’s novel, The Sorrows of Young Werther, which ended with the protagonist’s suicide. The novel was allegedly associated with a number of young men’s copycat suicides after its publication [84,85]. Studies suggest that vulnerability (eg, history of suicidal ideation or suicide attempt) and identification (eg, similar demographic backgrounds) with the featured character of a suicide story may contribute to this *contagion* effect [85]. Although there is a body of evidence on both the *Papageno* and *Werther* effects [86], to the best of our knowledge, this is the first study to consider how these may also apply to young people’s web-based communication about suicide with their peers. Despite interest from young people in consuming stories of lived experience for logical and privacy reasons, we opted not to include direct personal stories in the first iteration of our public campaign. However, all content still sought to instill hope and reinforce recovery.

### Campaign Format

Finally, in terms of campaign format, and akin to other health promotion research [87], young people in this study wanted the campaign to be delivered via short videos, animations, photographs, and images with text overlay. Animations, in particular, were popular among young people and have previously been described as “non-threatening, familiar, and accessible across age groups, cultures, and literacy levels” [88].

### Limitations

There are limitations to this study. The first relates to the representativeness of the sample. Most young people were recruited through youth advocacy programs in metropolitan Australia and, therefore, were a self-selected sample who had high levels of mental health literacy and ready access to health services. Thus, #chatsafe campaign materials may not necessarily be relevant or acceptable to all young people. Youth advocacy programs were selected as recruitment sources primarily for safety reasons, as we wanted to ensure that young people were engaged with mental health services that could provide ongoing support if necessary. The generalizability of the campaign may be further limited by the demographic characteristics of our sample, which do not reflect the general population. For example, almost one-half of our sample identified as nonheterosexual. As a result, the campaign might address specific, rather than general, needs. Despite this, the groups represented in our sample included young people who are frequently overrepresented in the suicide statistics (those experiencing mental ill-health, Aboriginal and Torres Strait Islander, LGBTIQA+, and/or CALD); hence, it was considered important to target them [89-92]. Second, a robust evaluation of the co-design process or campaign materials was not conducted, so as to prevent overburdening of the young people involved.

To improve generalizability and explore the reach and impact of the social media campaign, a trial targeting all Australian young people aged between 18 and 25 years is currently underway, and these results will be reported separately. Moreover, we are currently working with people from other diverse communities on adapting the guidelines and campaign materials for different cultural groups across the globe. The #chatsafe guidelines are also currently being translated into a number of additional languages.

### Conclusions

Despite significant government investment and a recent increase in research efforts [26,93,94], youth suicide rates continue to rise [1-3]; therefore, new approaches to youth suicide prevention are required. Mass media campaigns are gaining traction as a suicide prevention strategy [65], yet, to date, none focus specifically on young people or address the multiple ways in which they use social media to communicate about suicide. This study was the first internationally to co-design a social media campaign that aimed to facilitate safe peer-to-peer communication about suicide on the web. The co-design process led to the generation of valuable recommendations for the format and content of the campaign to boost its acceptability to young people. The adapted co-design process was found to be feasible, safe, and acceptable and highlights the importance of modifying methodology when undertaking a co-design process with young people in the suicide prevention arena. Finally, participating in the co-design process led to increased suicide literacy among young people. The #chatsafe social media campaign was rolled out across Australia. If effective, the campaign has the potential to better equip many young people worldwide to talk safely on the web about suicide.

## Appendix

Multimedia Appendix 1 Demographic characteristics of young people who participated in the co-design workshops (N=131).

Multimedia Appendix 2 Workshop evaluation results (N=131).

## Abbreviations

AI: artificial intelligence
CALD: culturally and linguistically diverse
LGBTIQA+: lesbian, gay, bisexual, transgender, intersex, queer/questioning, asexual, and other gender or sexual orientation
