# Supplementary material for: Developing a Suicide Prevention Social Media Campaign With Young People (The #Chatsafe Project): Co-Design Approach
Source: JMIR Ment Health. 2020 May 11;7(5):e17520. doi: 10.2196/17520 (PMC7248803; doi:10.2196/17520)
Supplement: Multimedia Appendix 1 [file mental_v7i5e17520_app1.docx]

**Multimedia Appendix 1, Table 1.** **Demographic characteristics of young people who participated in the co-design workshops (*N*=131).**

| Variable | | Values |
| --- | --- | --- |
| Age (years), mean (SD, range) | | 21.23 (1.90, 17-25) |
| **Gender, n (%)** | | |
|  | Male | 24 (18.3) |
|  | Female | 70 (53.4) |
|  | Transgender | 5 (3.8) |
|  | Other | 19 (14.5) |
|  | Prefer not to say | 1 (0.8) |
|  | Missing | 12 (9.2) |
| **Sexual orientation, n (%)** | | |
|  | Heterosexual | 65 (49.6) |
|  | Lesbian | 5 (3.8) |
|  | Gay | 8 (6.1) |
|  | Bisexual | 24 (18.3) |
|  | Questioning | 1 (0.8) |
|  | Other | 21 (16.0) |
|  | Missing | 7 (5.3) |
| **Aboriginal and/or Torres Strait Islander, n (%)** | | |
|  | No | 118 (90.1) |
|  | Yes | 13 (9.9) |
|  | Missing | 0 (0.0) |
| **English spoken at home, n (%)** | | |
|  | No | 22 (16.8) |
|  | Yes | 108 (82.4) |
|  | Missing | 1 (0.8) |
| **Bilingual or multilingual, n (%)** | | |
|  | No | 89 (67.9) |
|  | Yes | 41 (31.3) |
|  | Missing | 1 (0.8) |
| **Australian state or territory, n (%)** | | |
|  | Australian Capital Territory | 1 (0.8) |
|  | New South Wales | 18 (13.7) |
|  | Northern Territory | 0 (0.0) |
|  | Queensland | 3 (2.3) |
|  | South Australia | 21 (16.0) |
|  | Tasmania | 0 (0.0) |
|  | Victoria | 57 (43.5) |
|  | Western Australia | 31 (23.7) |
|  | Missing | 0 (0.0) |
| **Australian geographic area classification zone, n (%)** | | |
|  | Metropolitan | 111 (84.7) |
|  | Regional | 13 (9.9) |
|  | Rural | 1 (0.8) |
|  | Remote | 2 (1.5) |
|  | Missing | 4 (3.1) |
| **Experienced a mental health problem, n (%)** | | |
|  | No | 19 (14.5) |
|  | Yes | 110 (84.0) |
|  | Missing | 2 (1.5) |
| **Experienced suicidal ideation, n (%)** | | |
|  | No | 26 (19.8) |
|  | Yes | 103 (78.6) |
|  | Missing | 2 (1.5) |
| **Engaged in self-harm, n (%)** | | |
|  | No | 43 (32.8) |
|  | Yes | 86 (65.6) |
|  | Missing | 2 (1.5) |
| **Supported a friend experiencing a mental health problem, n (%)** | | |
|  | No | 3 (2.3) |
|  | Yes | 127 (96.9) |
|  | Missing | 1 (0.8) |
| **Supported a friend experiencing suicidal ideation, n (%)** | | |
|  | No | 18 (13.7) |
|  | Yes | 112 (85.5) |
|  | Missing | 1 (0.8) |
| **Exposed to suicide-related content on social media, n (%)** | | |
|  | No | 13 (9.9) |
|  | Yes | 116 (88.5) |
|  | Missing | 2 (1.5) |
| **Lost someone close to suicide, n (%)** | | |
|  | No | 68 (51.9) |
|  | Yes | 59 (45.0) |
|  | Missing | 4 (3.1) |

**This is a Multimedia Appendix to a full manuscript entitled “The #chatsafe Project: Co-designing a Suicide Prevention Social Media Campaign with Young People”.**
