# Supplementary material for: Developing a Suicide Prevention Social Media Campaign With Young People (The #Chatsafe Project): Co-Design Approach
Source: JMIR Ment Health. 2020 May 11;7(5):e17520. doi: 10.2196/17520 (PMC7248803; doi:10.2196/17520)
Supplement: Multimedia Appendix 2 [file mental_v7i5e17520_app2.docx]

**Multimedia Appendix 2, Table 2. Workshop evaluation results (*N*=131).**

| Variable | | | Values |
| --- | --- | --- | --- |
| **General skills and confidence** | | | |
|  | **Developed new skills, n (%)** | | |
|  |  | Disagree | 6 (4.6) |
|  |  | Agree | 98 (74.8) |
|  |  | Unsure | 27 (20.6) |
|  |  | Missing | 0 (0.0) |
|  | **Improved communication skills, n (%)** | | |
|  |  | Disagree | 9 (6.9) |
|  |  | Agree | 89 (67.9) |
|  |  | Unsure | 33 (25.2) |
|  |  | Missing | 0 (0.0) |
|  | **Improved interpersonal skills, n (%)** | | |
|  |  | Disagree | 9 (6.9) |
|  |  | Agree | 74 (56.5) |
|  |  | Unsure | 47 (35.9) |
|  |  | Missing | 1 (0.8) |
|  | **Improved self-confidence, n (%)** | | |
|  |  | Disagree | 17 (13.0) |
|  |  | Agree | 51 (38.9) |
|  |  | Unsure | 63 (48.1) |
|  |  | Missing | 0 (0.0) |
| **Supporting others** | | | |
|  | **Better equipped to provide emotional support to others,** **n (%)** | | |
|  |  | Disagree | 13 (9.9) |
|  |  | Agree | 71 (54.2) |
|  |  | Unsure | 46 (35.1) |
|  |  | Missing | 1 (0.8) |
|  | **Increased ability to educate others about online safety,** **n (%)** | | |
|  |  | Disagree | 11 (8.4) |
|  |  | Agree | 82 (62.6) |
|  |  | Unsure | 38 (29.0) |
|  |  | Missing | 0 (0.0) |
| **Suicide literacy** | | | |
|  | **Better understanding of how to communicate about suicide generally,** **n (%)** | | |
|  |  | Disagree | 11 (8.4) |
|  |  | Agree | 75 (57.3) |
|  |  | Unsure | 45 (34.4) |
|  |  | Missing | 0 (0.0) |
|  | **Better understanding of how to communicate about suicide safely on the Web, n (%)** | | |
|  |  | Disagree | 10 (7.6) |
|  |  | Agree | 90 (68.7) |
|  |  | Unsure | 31 (23.7) |
|  |  | Missing | 0 (0.0) |
|  | **Better understanding of how to identify and support others on the Web who may be at risk of suicide,** **n (%)** | | |
|  |  | Disagree | 13 (9.9) |
|  |  | Agree | 80 (61.1) |
|  |  | Unsure | 37 (28.2) |
|  |  | Missing | 1 (0.8) |
| **Acceptability** | | | |
|  | **The #chatsafe project was enjoyable,** **n (%)** | | |
|  |  | Disagree | 2 (1.5) |
|  |  | Agree | 126 (96.2) |
|  |  | Unsure | 3 (2.3) |
|  |  | Missing | 0 (0.0) |
|  | **The #chatsafe project was helpful,** **n (%)** | | |
|  |  | Disagree | 1 (0.8) |
|  |  | Agree | 112 (85.5) |
|  |  | Unsure | 17 (13.0) |
|  |  | Missing | 1 (0.8) |
|  | **The #chatsafe project was worthwhile, n (%)** | | |
|  |  | Disagree | 3 (2.3) |
|  |  | Agree | 119 (90.8) |
|  |  | Unsure | 8 (6.1) |
|  |  | Missing | 1 (0.8) |
|  | **Would recommend the #chatsafe project to a friend, n (%)** | | |
|  |  | Disagree | 2 (1.5) |
|  |  | Agree | 123 (93.9) |
|  |  | Unsure | 6 (4.6) |
|  |  | Missing | 0 (0.0) |
| **Safety** | | | |
|  | **The #chatsafe workshop made me feel upset,** **n (%)** | | |
|  |  | Disagree | 103 (78.6) |
|  |  | Agree | 9 (6.9) |
|  |  | Unsure | 18 (13.7) |
|  |  | Missing | 1 (0.8) |
|  | **The #chatsafe workshop made me feel suicidal,** **n (%)** | | |
|  |  | Disagree | 116 (88.5) |
|  |  | Agree | 8 (6.1) |
|  |  | Unsure | 6 (4.6) |
|  |  | Missing | 1 (0.8) |

**This is a Multimedia Appendix to a full manuscript entitled “The #chatsafe Project: Co-designing a Suicide Prevention Social Media Campaign with Young People”.**
